# Ocular safety of cationic emulsion of cyclosporine in an in vitro corneal wound-healing model and an acute in vivo rabbit model

**Published:** 2012-08-08

**Authors:** Hong Liang, Christophe Baudouin, Philippe Daull, Jean-Sébastien Garrigue, Françoise Brignole-Baudouin

**Affiliations:** 1 INSERM, Paris, France; 2 UPMC Univ Paris 06, Institut de la Vision, Paris, France; 3 CNRS, Paris, France; 4 Centre Hospitalier National d’Ophtalmologie des Quinze-Vingts, Paris, France; 5 Assistance Publique - Hôpitaux de Paris Hôpital Ambroise Paré, Service d’Ophtalmologie, Boulogne-Billancourt, France; 6 Université Versailles Saint-Quentin-en-Yvelines, Versailles, France; 7 Novagali Pharma, Evry, France; 8 Université Paris Descartes, Sorbonne Paris Cité, Faculté des Sciences Pharmaceutiques et Biologiques, Laboratoire de Toxicologie, Paris, France

## Abstract

**Purpose:**

Topical preparations of cyclosporine (CsA) are common therapeutics for the treatment of dry eye. However, they are not devoid of side effects, such as allergy and irritation. The present study aimed at evaluating the safety profile of a new CsA formulation in cationic emulsion (CEm) in vitro with a dynamic corneal wound healing assay using human corneal epithelial (HCE) cells, and in vivo in a rabbit acute toxicity model.

**Methods:**

Three different CsA formulations were tested: 1) 0.05%CsA-CEm, 2) commercial 0.05%CsA-Anionic emulsion (CsA-AEm, Restasis^®^), and 3) 0.05%CsA-Oil solution. Phosphate buffered saline (PBS) was used as negative control and 0.02% benzalkonium chloride (BAK) as the toxic control. In vitro, a wound was created by scratching through a confluent HCE cell layer and exposed 30 min to 1/10 dilutions of the different formulations. Cytotoxicity, cell migration, and proliferation were performed to analyze the recovery at days 1, 2, and 3. In vivo, the eye drops were applied to rabbit eyes 15 times at 5-min intervals. The ocular surface structures were examined with a slit-lamp and by corneal in vivo confocal microscopy (IVCM) for detailed examination of corneal epithelium, stroma, limbus, and conjunctiva-associated lymphoid tissue (CALT) structures.

**Results:**

The in vitro study confirmed that a 0.02% BAK solution delayed the corneal healing process (−57%) by severely damaging the remaining HCE cells. The other formulations maintained a normal healing rate with a similar behavior for CsA-CEm, CsA-AEm, and PBS with no significant differences (at D3, 66%–74% closure). In the rabbit, 0.02%BAK showed the highest toxicity, inducing redness, chemosis with damaged corneal epithelium, and inflammatory cell infiltrations. CsA-AEm and CsA-Oil induced moderate infiltrations of inflammatory cells around the CALT. CsA-CEm presented the lowest toxicity with patterns similar to PBS.

**Conclusions:**

The combination of these in vitro and in vivo models evaluated the tolerance/cytotoxicity and the dynamic wound healing potential of CsA in different formulations. While CsA-AEm, CsA-CEm, and CsA-Oil are generally well tolerated, only CsA-CEm appeared to maintain the HCE cells’ normal healing rate in vitro and low levels of inflammation in vivo.

## Introduction

Cyclosporine A (CsA) is a potent immunosuppressive agent that inhibits the proliferation and action of T lymphocytes by blocking the transcription of cytokines [1]. Systemic CsA has been used in organ transplantation for the past few decades, and topical CsA has been used for the treatment of ocular surface diseases since the early 1980s with considerable success. Systemic and topical hospital CsA preparations were used to treat severe ocular surface diseases including uveitis, cornea transplant rejection, severe vernal keratoconjunctivitis (VKC), necrotizing scleritis, Behçet syndrome, high-risk corneal transplantation, and adenoviral kerato-conjunctivitis [2-6]. In 2003, an ophthalmic anionic emulsion containing 0.05% CsA (Restasis^®^, Allergan, Irvine, CA, USA) was approved by the United States Food and Drug Administration (FDA) to increase tear production in patients whose tear production is presumed to be suppressed due to ocular inflammation associated with keratoconjunctivitis sicca (dry eye syndrome) [7,8].

Many clinical reports have presented the efficacy and the safety of long-term treatment of patients with moderate to severe dry eye disease with 0.05%CsA ophthalmic emulsion (Restasis^®^) [9]. In these patients, CsA improves the symptoms and signs of dry eye, effectively controlling inflammation [10]. However, while hospital preparations of CsA are commonly used for the treatment and management of VKC [11], repeated instillations of 0.05% CsA anionic emulsion (Restasis^®^) failed as a steroid-sparing agent in VKC [12].

CsA instillations can induce some discomfort, such as stinging or burning sensations following the installations and redness. However, despite these discomforts, treatments of chronic dry eye disease with 0.1% CsA ophthalmic emulsion for 1–3 years were found to be safe, well tolerated, and not associated with systemic side effects [13]. For example, in a 12-week and 1-year clinical safety study in dry eye patients, the most common adverse event reported for cyclosporine emulsion was ocular burning sensation with no serious drug-related adverse event [14]. Other eye drops known for their itchy, sandy, and foreign body sensations upon instillations are those containing preservative concentrations of benzalkonium chloride (BAK), such as preserved antiglaucoma therapies that contain up to 0.02% BAK.

CsA is a lipophilic compound readily soluble in oil: in pure olive, corn, castor oil, minera oil, or medium-chain triglycerides for hospital preparations [15] or in emulsified mineral oil for Restasis^®^, a CsA-anionic emulsion (CsA-AEm, Restasis^®^; Allergan Inc., Irvine, CA). A novel formulation, based on the Novasorb^®^ technology [16], a cationic emulsion suitable for the solubilization of lipophilic drugs, was developed with either CsA or latanoprost by Novagali Pharma (Evry, France) [17]. Preclinical data of this CsA-cationic emulsion (CsA-CEm) demonstrated that it delivered CsA to the cornea and conjunctiva very efficiently following single and repeated instillations in the rabbit [18]. Phase II clinical trials and an ongoing Phase III clinical trial with the preservative-free CsA-CEm (Cyclokat^®^) demonstrated the safety and good tolerance of this new CsA formulation.

To further evaluate this newly developed CsA-CEm, we undertook a series of experiments in which CsA-CEm, CsA-AEm, and CsA-Oil were compared to negative (phosphate buffered saline, PBS) and positive (0.02%BAK solution) controls with new and sensitive in vivo and in vitro approaches. Our purpose was to evaluate 1) the influence of CsA on the reepithelialization of a scraped cultured human corneal epithelial cell monolayer and 2) the tolerance of the CsA-CEm on rabbit ocular surface using an established acute irritation model.

## Methods

### Human corneal epithelium cell culture

A human corneal epithelial cell line (HCE, RCB-1384; Riken Cell Bank, Tsukuba, Japan) was cultured under standard conditions using a 1:1 mixture of Dulbecco's modiﬁed Eagle medium (DMEM) and Nutrient Mixture F12 supplemented with 10% fetal bovine serum (FBS), 2 mM l-glutamine, and antibiotics (penicillin, streptomycin). Cells were seeded in six-well culture dishes at a density of 100,000 cells per well and kept at 37 °C for 24 h. HCE cells were grown to 90%–95% confluence before the experiments.

### In vitro wound-healing assay and eye drop incubations

The different solutions tested were sterile phosphate-buffered saline (PBS), CsA in cationic emulsion (CsA-CEm; Novagali Pharma, Evry, France), CsA anionic emulsion (CsA-AEm, Restasis^®^; Allergan), CsA in oil (CsA-Oil; medium-chain triglycerides, Mygliol^®^ 812; IMCD, Saint-Denis, France), and a 0.02% BAK solution, the highest concentration of the commonly used eye drop preservative. All CsA formulations are preservative-free, with CsA-AEm, and CsA-CEm having physiologic pH and osmolality. A standardized wound was created by scratching the HCE cell monolayer with a sterile micropipette tip under an inverted microscope. The cells were then incubated 30 min with 1:10 dilutions (in cell culture medium) of the aforementioned test items as described in a previous study that evaluated different commercial eye drops in the cell culture [19,20]. After 30 min, the incubation medium containing the different 1:10 eye drop dilutions was discarded, the cell culture plate washed to remove any resuspended cells and replaced with fresh cell culture medium.

### Wound-healing analyses: Wound distances, percentage of closure

At Day (D) 0, D1, D2, and D3, each well was photographed at 200× magnification with an inverted microscope (Leica DMIRB; Leica, Heidelberg, Germany) equipped with a digital camera (Coolpix 5000; Nikon, Tokyo, Japan). The wound distances (WDs) were measured with a framing graticule that provides precise length measurement in three different areas alongside the scraping, corresponding to the superior, middle, and inferior WD. The final calculated WD was the mean value of these three measured lengths. In case of cytotoxicity inducing cell detachment, the WD was measured between the edges of the first cell aggregates. The WD at D0, before the incubation with the eye drop dilutions, was used as the reference to calculate the percentage of closure over time. All the experiments were repeated three times, and the final calculated WD was the mean of three experiments.

### Animals and acute instillation model

All experiments were conducted in accordance with the Association for Research in Vision and Ophthalmology’s (ARVO) Statement for the Use of Animals in Ophthalmic and Vision Research. The research was approved by the local ethics committee for animal experimentation of the Faculty of Pharmaceutical and Biologic Sciences, Paris Descartes University. All rabbits used for the in vivo experiment weighed 2–2.5 kg (Cegav, St. Mars-d'Egrenne, France). The experimental procedure follows a previously published protocol [17,21]. Briefly, all the solutions (50 µl per instillation) were instilled 15 times at 5-min intervals, i.e., the last instillation was performed 75 min after the first instillation.

### Clinical observation and Draize test

The rabbit ocular surface was evaluated at 75 min (immediately after the last instillation), H4 and D1. The modified Draize test scale defined in a previous study [17] was used for the evaluation of conjunctival hyperemia, chemosis, and purulent secretions following the repeated installations with the different eye drop preparations [17,21]. We evaluated the degree of redness, chemosis, and tearing of the conjunctiva; the degree and area of cornea opacity; and the increased prominence of folds and congestion of the iris. The possible maximum total score was 110 (conjunctiva=20, cornea=80, iris=10).

### IVCM analysis and IVCM score

A laser-scanning IVCM Heidelberg Retina Tomography (HRT) II/Rostock Cornea Module (RCM; Heidelberg Engineering GmbH, Heidelberg, Germany) was used to examine the entire ocular surface (cornea, conjunctiva, limbus, and the conjunctiva-associated lymphoid tissue: CALT) and quantify the changes and the cytotoxicity with the previously described IVCM scale system [17]. Scores were obtained from five zones: the superficial epithelium, basal epithelium, and anterior stroma of the cornea, limbus, and conjunctiva-associated lymphoid tissue (CALT). Cell morphology and nuclear aspects were evaluated, and the number of infiltrating inflammatory cells (lymphocytes, polymorphonuclear cells, or dendritic-like cells) was assessed by using the Cell Count® program (Heidelberg Engineering GmbH) associated with the HRT II/RCM.

## Results

### Effects of the different treatments on wound closure in an in vitro HCE cell scraping assay

**Figure 1:**
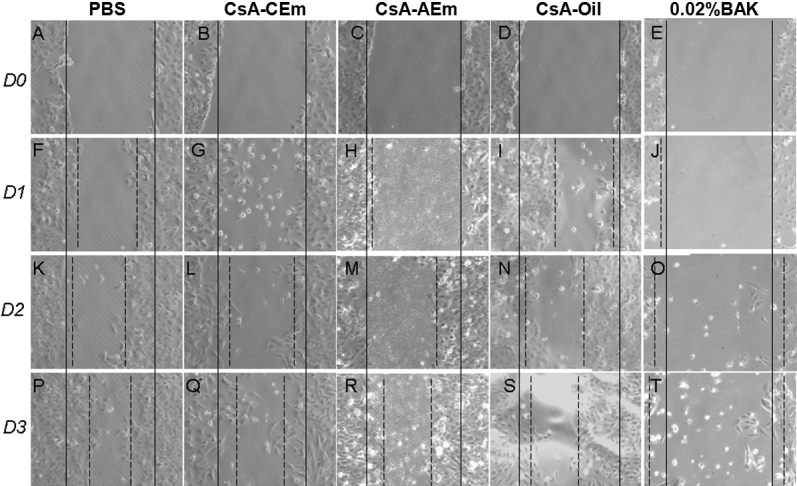
HCE wound healing evaluation from Day 1 to Day 3. Effects of PBS (control; column 1), CsA-CEm (column 2), CsA-AEm (column 3), CsA-Oil (column 4), and 0.02% BAK solution (column 5) on HCE cell wound closure at D0, D1, D2, and D3. Closure of the wound is observed for PBS and the three CsA groups at D1 and progresses until D3. 0.02% BAK treatment did not result in observable wound closure; on the contrary, it induced cell death and widening of the wound. The solid lines indicate the initial WD, and the dashed lines indicate the new edge positions at the different time points.

After HCE cells were scraped and incubated with PBS, CsA-CEm, CsA-AEm, CsA-Oil, or 0.02%BAK, the pace of the wound closure was measured at D1, D2, and D3. At D0 (i.e., before treatment), no differences in the width of the scraped area were noted, demonstrating the repeatability of the scraping procedure (see line 1 A–E (D0) in Figure 1).

At D1, following exposure to 0.02%BAK (Figure 1J), numerous dark, round, detached cells were observed near the edges, with the widening of the scraped area (wound). In contrast, cells appeared to be normal following treatments with PBS (Figure 1F), CsA-CEm (Figure 1G), CsA-AEm (Figure 1H), or CsA-Oil (Figure 1I), with narrowing of the scraped area already noticeable. At D2, the healing had progressed in the plates treated with PBS, CsA-CEm, CsA-AEm, and CsA-Oil (Figure 1, line 3: K–N), whereas at the same time, 0.02% BAK-treated cells (Figure 1O) did not present any tendency toward recovery, with a progression of the widening of the scraped area. At D3, in 0.02%BAK-treated wells it was difficult to measure the distance between the edges (Figure 1T), because HCE cells near the edges detached from the dish with numerous dark, hyper-reflective dead cells freely flowing inside the scraped area. In contrast, the three CsA formulation-treated wells presented a scraped area with approximately 60% closure efficacy (Figure 1Q–S) and performed just as well as PBS-treated cells (Figure 1P). Interestingly, from D1 to D3 in CsA-AEm-treated wells, tiny dark spots or particles were noted on the bare plastic of the scraped area. However, their presence had no incidence on the duration of the wound healing process.

### In vitro percentage of wound closure over time

**Figure 2:**
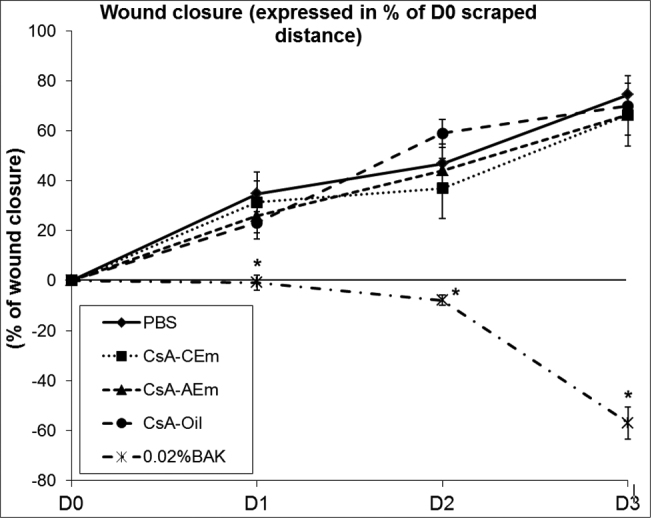
Wound closure from D1 to D3 (expressed as a percentage of the D0 WD) after the different treatments. The positive control PBS and the three CsA formulations have nearly identical wound closure rates of reepithelialization from D1 to D3. The negative control, 0.02%BAK, increased wound size and inhibited the healing process from D1 to D3. * p<0.0001–0.02 compared to the other four groups at the corresponding times.

The calculated wound closure percentages from D1 to D3 are presented in Figure 2. As for the control, the three CsA formulations did not delay the healing process. At D3, no significant differences in the wound closure of the CsA formulation-treated or PBS-treated groups were noted, with closure reaching 66.46%, 66.37%, and 70.03% for CsA-CEm, CsA-AEm, and CsA-Oil, respectively, and 74.54% for PBS. The 0.02% BAK solution significantly delayed the wound healing process (p<0.0001–0.02 when compared to the other groups at the different time points).

### In vivo clinical observations and Draize test

**Figure 3:**
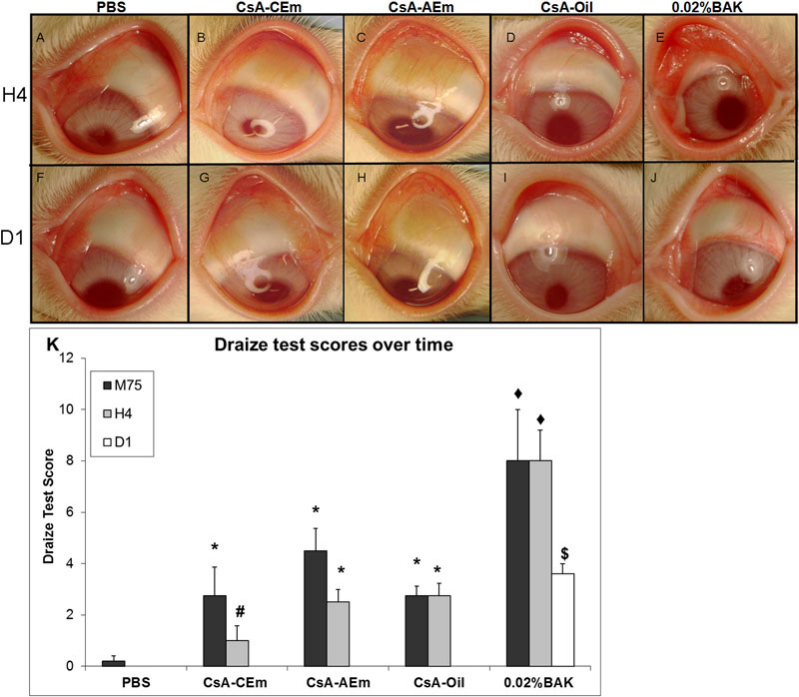
Microphotographs of the treated eyes 4 h after the 15 instillations and Draize test scores. Representative microphotographs of eyes treated with PBS (**A**, **F**), CsA-CEm (**B**, **G**), CsA-AEm (**C**, **H**), CsA-Oil (**D**, **I**), and 0.02% BAK (**E**, **J**) at the 4 h (H4) and 1 day (D) time points. The Draize test scores were calculated at each time point and reported in **K**. At the H4 time point, 0.02% BAK induced diffuse hyperemia, chemosis, and purulent secretions of the conjunctiva that receded at D1. Compared to PBS-treated eyes, the three CsA formulations induced slight to moderate hyperemia with neither purulent secretions nor chemosis at the three time points. At D1, only 0.02% BAK-treated eyes presented hyperemia. * p<0.02 compared to PBS and p<0.004 compared to 0.02% BAK. # p<0.01 compared to CsA-AEm, CsA-Oil, and 0.02% BAK. ◆ p<0.0001 compared to PBS. $ p=0.0003 compared to PBS, Cs-CEm, CsA-AEm, and CsA-Oil groups.

The repeated instillations (15 instillations over 75 min), with the three CsA formulations resulted in hyperemia of the ocular surface with Draize test scores (DTS) ranging from 2 to 5 immediately after the last instillation. At H4, while PBS-treated animals (Figure 3A) presented a normal ocular surface, and the 0.02% BAK-treated animals (Figure 3E) had an ocular surface with obvious signs of toxicity – hyperemia, chemosis, and purulent secretions – the three CsA formulation-treated animals still presented slight hyperemia with neither purulent secretions nor chemosis (Figure 3B-D). At D1, PBS-treated (Figure 3F) and CsA formulation-treated animals (Figure 3G-I) showed normal aspects in their treated eyes. In contrast, 0.02%BAK-instilled eyes still presented slight hyperemia of the bulbar conjunctiva (Figure 3J).

Draize test scores are presented in (Figure 3K). The 0.02%BAK-treated eyes had the highest score (DTS=8, 8, and 3.8 at M75, H4, and D1, respectively) among the treatment groups at the different time points. At 75 min, the CsA formulation-treated eyes presented moderate irritation scores (DTS ranging from 2.6 to 4.5), which were higher (p<0.02) than those observed for PBS-treated eyes (DTS=0.8) and lower than in BAK-instilled eyes (p<0.004). No difference in DTS was found among the three CsA formulation-treated groups at the 75-min time point. At H4, the DTS of CsA-CEm-treated eyes was significantly lower than with the two other CsA formulations (p<0.01) and the 0.02% BAK treatments (p<0.01). At D1, no irritation DTS was measurable in PBS- and CsA formulation-treated animals; only 0.02% BAK treatment induced hyperemia that was still noticeable (DTS=3.6, p<0.0003 when compared to the other treatment groups). At D1, PBS- and CsA formulation-treated eyes appeared to be not different from untreated eyes.

### IVCM images and IVCM scale analyses

**Figure 4:**
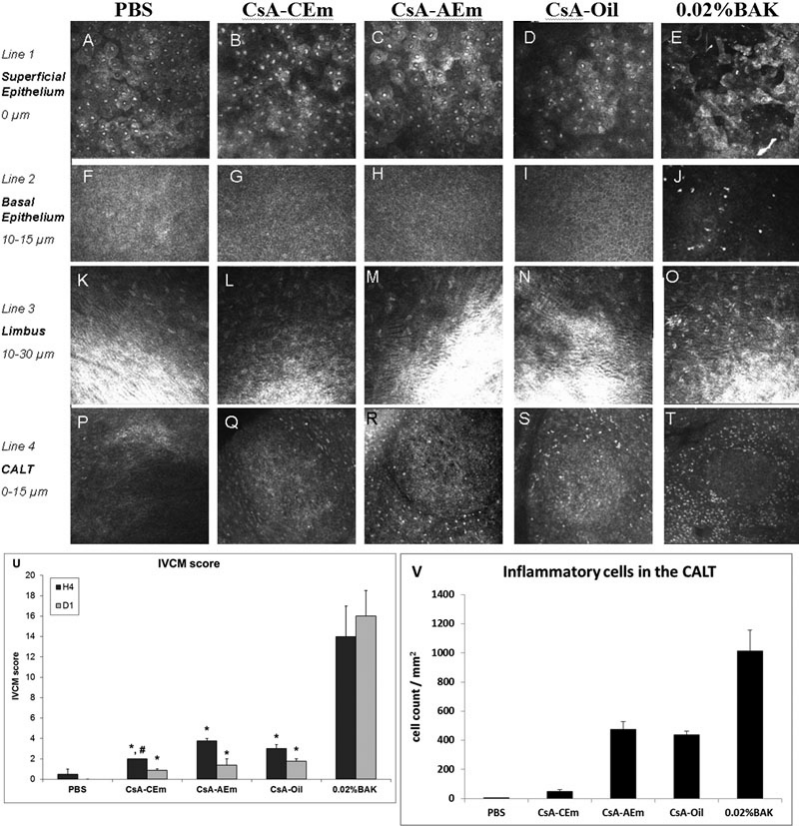
IVCM images and IVCM scale analyses. HRT II IVCM images of rabbit ocular surface 4 h after the repeated instillations of PBS (column 1), CsA-CEm (column 2), CsA-AEm (column 3), CsA-oil (column 4), and 0.02%BAK (column 5). Results are shown for the superficial epithelium (line 1), basal epithelium (line 2: 10–15 μm from the superficial epithelium layer), limbus (line 3: 10–30 μm from the superficial epithelium layer), and CALT (line 4: 0–15 μm from dome layer). U: IVCM score, and V: inflammatory cell counts in the CALT for the different treatments. BAK 0.02%-treated eyes presented the greatest damage (superficial epithelium disorganization and significant inflammatory cell infiltration in both basal epithelium and limbus). Extremely heavy infiltration of inflammatory cells inside and outside the CALT structure can also be observed. The three CsA formulations induced some inflammatory cell infiltration mainly around the CALT structure without alteration of the corneal structure. (Images: 400 µm×400 µm). Note the similarity of P, Q, R, and S.* p≤0.03 compared to PBS and p<0.0001 compared to 0.02%BAK. # p<0.05 compared to CsA-AEm.

The treated eyes’ microstructures were analyzed by IVCM following the instillations with the different CsA formulations at 4 h: representative pictures of the superficial epithelium (line 1), basal epithelium (line 2), limbus (line 3), and CALT structures (line 4) are presented in Figure 4.

#### Superficial epithelium

Repeated instillations of PBS did not alter the superficial epithelium (Figure 4A), while 0.02% BAK (Figure 4E) induced substantial damage such as partial desquamation of epithelial cells, irregular cell shape with abnormal reflectivity patterns, cell swelling, cell border loss and inflammatory cell infiltrates. In contrast, with the three CsA formulations the superficial epithelium showed an almost normal aspect with a regular polygonal mosaic appearance with bright and reflective nuclei (Figure 4B-D). A slight shrinkage of epithelial cells in CsA-AEm-treated animals was observed.

#### Basal epithelium

PBS- and the CsA formulation-treated eyes presented a normal basal epithelium with very little inflammatory cell infiltration (<5 cells/mm^2^; Figure 4F-I). The 0.02% BAK treatment (Figure 4J) induced considerable inflammation, with the infiltration of 139±19 inflammatory cells/mm^2^.

#### Limbus

The limbus was normal in the animals treated with the three CsA formulations (Figure 4K-N). The 0.02% BAK solution induced a limbic inflammatory cell infiltration with the hyper-reflective spots (Figure 4O) that characterize the inflammatory cells.

#### Conjunctiva-associated lymphoid tissue observation

The IVCM allowed us to observe the lymphoid follicles of the CALT throughout the bulbar conjunctiva and partially in the fornix. After repeated instillations of PBS (Figure 4P), the rabbit CALT observations revealed that the CALT was normal with fewer than 5 inflammatory cells/mm^2^ around the follicle. The 0.02% BAK solution (Figure 4T) induced violent inflammation with tremendous inflammatory cell infiltration (possibly polymorphonuclear cells and lymphocytes) surrounding the CALT structure, as illustrated by substantial inflammatory cell attraction and trafficking (1,012±145 inflammatory cells/mm^2^). Interestingly, CsA-CEm treatment (Figure 4Q) induced a very low level of inflammation, with a low number of inflammatory cells present in the CALT (50±12 inflammatory cells/mm^2^), while with the two other CsA formulations (Figure 4R,S) treatment resulted in moderate inflammation, with 475±52 inflammatory cells/mm^2^ for CsA-AEm and 436±26 inflammatory cells/mm^2^ for CsA-Oil (Figure 4V).

The plotted scores of the IVCM scale analysis (Figure 4U) clearly showed that repeated instillations of 0.02% BAK induced major damage to the ocular surface of the treated rabbits. The three CsA formulation treatments resulted in IVCM scores indicative of slight toxicity. These scores were higher than with PBS but much lower than with 0.02% BAK (p≤0.03 when compared to PBS and p<0.0001 when compared to 0.02% BAK). At H4, CsA-CEm presented a lower IVCM score than CsA-AEm (p<0.05). At D1, there was no significant difference between the three CsA formulations’ IVCM scores. However, these scores remained significantly higher than for PBS (p<0.03). The three CsA formulations’ IVCM scores were much lower than the 0.02%BAK score (p<0.0001).

## Discussion

Although the positive impact of CsA has been clearly demonstrated for the treatment of severe chronic ocular surface pathologies, little information is available on the different alternative CsA formulations, and the mechanism underlining its irritating effects remains unclear. In this study, three CsA formulations were tested with two different models that we had developed previously and used for toxicological evaluation of eye drops: i.e., the in vitro dynamic wound healing model in HCE cells [22] and the acute toxicity rabbit model [17]. With both models, the three CsA formulations presented very good safety profiles. The three CsA formulations did not delay the wound healing process, with similar wound closure rates over time, even though tiny dark particles were observed in CsA-AEm-treated wells that might be associated with cellular debris. The nature of these small particles was not evaluated any further. Repeated instillations with the three CsA formulations were well tolerated by the rabbit ocular surface, with the receding at D1 of the slight redness visible soon after the end of the instillations. The IVCM analysis of the ocular surface did not reveal any significant alterations in the eyes treated with the three CsA formulations: the corneal epithelium was almost normal without any significant inflammatory cell infiltration in both the basal epithelium and limbus. On the contrary, the CALT constitutes an ocular surface entity in itself whose detailed analysis by IVCM may help characterize the inflammatory and toxicologic response of different treatments [23]. Interestingly, a moderate CALT activation was observed after the repeated instillations with CsA-AEm and CsA-Oil, which was only 2.1 times lower than the one observed with 0.02% BAK. CsA-CEm-treated eyes had a slight CALT activation that was close to that observed with PBS. However, the inflammatory cell infiltration within the CALT completely disappeared at D1 for the three CsA formulations. It is interesting to note that repeated instillations of 0.02% BAK induced inflammatory cell infiltration in the CALT that is almost equivalent to that observed after the instillation of LPS, for example, as seen in Liang and collaborators [23], and that classic CsA formulations induced inflammatory cell infiltration in the CALT within the range of the infiltration induced by tumor necrosis factor alpha (TNFα). Globally, the three CsA formulations were well tolerated without any obvious signs of toxicity. These results are consistent with other studies published that demonstrate that CsA eye drop preparations are generally well tolerated. For example, in 12-week and 1-year clinical safety studies in dry eye patients, the most common adverse event reported with the use of ophthalmic CsA emulsion (CsA-AEm) was ocular burning. No serious drug-related adverse events occurred [14]. In animal models, neither ocular nor systemic toxicity was observed following long-term ocular administration of CsA at concentrations up to 0.4% and given as many as six times daily for 6 months in rabbits or for 1 year in dogs [14].

The successful and safe use of CsA in the ophthalmological field now encompasses many other fields, such as laser-assisted in situ keratomileusis (LASIK) surgery [24] and thyroid orbitopathy-related dry eye [25]. In fact, as the first FDA-approved therapy for KCS, CsA significantly increased conjunctival goblet cell density [26,27]. CsA increased goblet cell density [28,29] and induced nerve growth factor (NGF) expression [30] and it significantly reduced the activated lymphocytes [31]. In addition, after topical CsA eye drop instillation, the active agent is limited to the ocular surface tissues without penetrating and influencing intraocular structures. Ben Ezra et al. [32-34] have studied the ocular penetration of CsA in human, rabbit, and rat eyes. After a local treatment with 2% CsA in humans, no detectable levels of CsA were found in the blood, saliva, aqueous humor, or vitreous. In rabbits, instillation of 2% CsA in olive oil resulted in high concentrations of the drug in the cornea and conjunctiva with no detectable levels found within the intraocular structures.

Restasis^®^ has demonstrated that ophthalmic preparations of CsA were effective for the management of dry eye; however, in clinical practice the main CsA limitation is the discomfort sensation at instillation, which may in the most severe case lead to treatment cessation. Little is known on the mechanisms of such frequent intolerance and the respective roles of either CsA or its vehicle’s constituents (i.e., the excipients). Preclinical and clinical data do not satisfactorily answer this question, and we hypothesize that the ocular burning sensation might, at least in part, be associated with noticeable ocular structure disorganization. In an attempt to measure the impact of various CsA formulations on the ocular surface, in the present study we used a combination of in vitro and in vivo models that evaluate the safety and tolerance of repeated instillations of various CsA formulations, including a new CsA-cationic emulsion. The cytotoxicity of eye drops is usually evaluated with in vitro and in vivo models using normal, healthy, unstressed cells, tissues or animals. However, since CsA eye drop preparations are generally proposed to patients with KCS or other ocular surface pathologies, these simple tests did not adequately measure the impact of CsA on damaged ocular surfaces. Thus, this scraping model, which appropriately mimics the diseased ocular epithelium combined with the acute in vivo toxicity model that mimics long-term use of the drug over time, they both may help elucidate the effect of CsA eye drop preparations over time [22].

Our results demonstrate that CsA in cationic emulsion was safe and well tolerated, without impairing the physiologic functions of corneal epithelium in both an in vitro wound healing model and an in vivo acute toxicity rabbit model. The other two CsA formulations (CsA-AEm and CsA-Oil) were also well tolerated, even though their repeated instillations resulted in a relatively substantial inflammatory cell infiltration in the CALT. However, it is not possible to link this observation to the burning sensation induced by CsA ocular preparations. The moderate physical reaction upon instillation with CsA-AEm, when compared to 0.02%BAK, illustrated by the moderate cell shrinkage seen on panel C (Figure 4), most likely explains a nociceptive response of the cornea. The numerous pain-sensitive nerves present in the cornea might be activated by such slight cellular changes. The cationic emulsion formulations have been shown to reduce the toxicity of irritating molecules such as aliphatic chain analogs of benzalkonium chloride [21]. Cationic emulsions of CsA and latanoprost are currently under clinical development in KCS and glaucoma treatment, respectively, where they have demonstrated their good tolerance [35]. Compared to the anionic emulsion, cationic emulsion has a better bio-affinity for the negatively-charged ocular surface, which were found to be well tolerated after 15 instillations in rabbit and able to decrease the toxicity of BAK [21]. Our study showed that CALT could be a sensitive anatomic structure for the evaluation of subclinical cytotoxicity. It would be interesting to characterize the protective effects brought by CsA-CEm in the future by identifying inflammatory markers in CALT, such as CD45, CD4, or CD8.

In conclusion, this study demonstrated that CsA-CEm was as well tolerated as the other CsA formulations, with a significantly lower level of inflammatory cell infiltrates around the conjunctival lymphoid tissues with lower signs of irritation. It did not inhibit the wound healing process of cultured HCE cells, which is promising for the long-term treatment of the sensitive damaged ocular surface of patients having dry eye disease. The study also emphasizes the importance of the vehicle for the delivery of ocular CsA, as demonstrated by the seemingly pro-inflammatory effect of the oil or vehicle composition for CsA-Oil or CsA-AEm, for the treatment of allergic diseases such as VKC, and confirms the reliability of the use of CALT analysis for the evaluation of the safety/toxicity of new eye drop formulations. This newly developed cyclosporine formulation in cationic emulsion could therefore become a very good alternative to existing CsA formulations with an improved safety profile and potentially an improved benefit for patients having allergic ocular diseases.
